# Doffing Corridor: Establishing Expedited High-Volume Doffing With Exposure Reduction to Contaminated Personal Protective Equipment (PPE) in a COVID-19 Hospital in India

**DOI:** 10.7759/cureus.35529

**Published:** 2023-02-27

**Authors:** Rashmi Ranjan Guru, Sukhpal Singh, Navin Pandey, Manisha Biswal, Ritesh Agarwal, Inderpaul S Sehgal, Vipin Koushal, Girija S Mohanty

**Affiliations:** 1 Medicine, Post Graduate Institute of Medical Education and Research, Chandigarh, IND; 2 Hospital Administration, Post Graduate Institute of Medical Education and Research, Chandigarh, IND; 3 Medical Microbiology, Post Graduate Institute of Medical Education and Research, Chandigarh, IND; 4 Pulmonary Medicine, Post Graduate Institute of Medical Education and Research, Chandigarh, IND; 5 Obstetrics and Gynaecology, Post Graduate Institute of Medical Education and Research, Chandigarh, IND

**Keywords:** satisfaction in doffing corridor, doffing room, time taken for doffing, risk of infection, health care workers, errors in steps of doffing, covid-19 infection, doffing corridor

## Abstract

Background

Considering the virulent nature of the COVID-19, the safety of healthcare workers (HCW) became a challenge for hospital administrators. Wearing a personal protective equipment (PPE) kit, called donning, which can be easily done by the help of another staff. But correctly removing the infectious PPE kit (doffing) was a challenge. The increased number of HCWs for COVID-19 patient care raised the opportunity to develop an innovative method for the smooth doffing of PPEs.

Objective

We aimed to design and establish an innovative PPE doffing corridor in a tertiary care COVID-19 hospital during the pandemic in India with a heavy doffing rate and minimize the COVID-19 virus spread among healthcare workers.

Methodology

A prospective, observational cohort study at the COVID-19 hospital, Postgraduate Institute of Medical Education and Research (PGIMER), Chandigarh, India, was conducted from July 19, 2020, to March 30, 2021. The time taken for PPE doffing process of HCWs was observed and compared between the doffing room and doffing corridor. The data was collected by a public health nursing officer using Epicollect5 mobile software and Google forms. The following parameters, like grade of satisfaction, time and volume of doffing, the errors in the steps of doffing, rate of infection, were compared between the doffing corridor and the doffing room. The statistical analysis was done by the use of SPSS software.

Result

'Doffing corridor' decreased the overall doffing time by 50% compared to the initial doffing room. The doffing corridor solved the purpose of accommodating more HCWs for PPE doffing and an overall saving of 50% time. Fifty-one percent of HCWs rated the satisfaction rate as Good in the grading scale. The errors in the steps of doffing that occurred in the doffing process were comparatively lesser in the doffing corridor. The HCWs who doffed in the doffing corridor were three times less likely to get self-infection than the conventional doffing room.

Conclusion

Since COVID-19 was a new pandemic, the healthcare organizations focused on innovations to combat the spread of virus. One of these was an innovative doffing corridor to expedite the doffing process and decrease the exposure time to the contaminated items. The doffing corridor process can be considered at a high-interest rate to any hospital dealing with infectious disease, with high working satisfaction, less exposure to the contagion, and less risk of infection.

## Introduction

Severe acute respiratory syndrome coronavirus 2 (SARS-CoV-2), the causal agent of COVID-19, has become a global threat since its emergence. The World Health Organization (WHO) declared COVID-19 a pandemic on 20th January 2020 [1]. The top organizations like WHO, Center for Disease Control and Prevention (CDC), and the government played a significant role in facing the pandemic [2]. In a healthcare setup, proper use of a personal protective equipment (PPE) kit was essential for preventing infection in hospitals [3]. During this pandemic, our setup was transformed from a general hospital to an infectious disease hospital [4]. The process of wearing the components of PPE was called donning, and the removal of PPE was called doffing [5]. The designated area for disposing of contaminated PPE items was called a doffing area [6]. Doffing of the PPE kit in a sequential manner was done to prevent infection from contagion under real-time monitoring [7]. Here we provided descriptive details about the doffing corridor design, planning, and execution. The average time taken for doffing was much longer, increasing the waiting time; this problem led us to plan for a faster doffing technique at COVID-19 hospital, Postgraduate Institute of Medical Education and Research (PGIMER).

In the study, we aimed to design a doffing corridor to accommodate high-volume doffing with exposure reduction to contaminated PPE in a 300-bed COVID-19 hospital.

## Materials and methods

Study type

A prospective, observational cohort study was done at the COVID-19 hospital facility of PGIMER, Chandigarh, India, from July 19, 2020, to March 30, 2021.

The study participants were considered on a voluntary participation basis and were questioned by using a semi-structured questionnaire via Google forms and Epicollect 5 mobile software by a public health nurse. Demographic factors (age, sex, and occupation) and medical history were collected from all the participants before their duty [8]. The healthcare workers (HCWs) with chronic diseases and those who developed COVID-19 symptoms within five days of starting their duty in COVID-19 area, were excluded from the study.

In this prospective study, the doffing events were observed during the eight months period, where HCWs did daily six hours of duty for a period of seven days. These doffing events were done by the HCWs in doffing rooms and in doffing corridors as per their own choice. The time for doffing event was collected by real-time surveillance camera in the doffing room and the doffing corridor and compared. Post doffing corridor usage, the overall doffing satisfaction of the HCWs was assessed based on their reviews in a four-point grading system with subjective parameters like Excellent, Very Good, Good, and Poor. The errors in the steps of doffing event were collected from a real-time surveillance camera and recorded manually. The errors in the steps of doffing event were compared between the doffing corridor and the doffing room and presented in percentage. Descriptive statistics were used to identify the risk factors associated with doffing process. Odds ratio was calculated by taking the two groups of HCWs who doffed in the doffing corridor and the doffing room by considering a 95% Confidence Interval (CI) and a p-value of <0.05. The statistical analysis was done using SPSS software (IBM Corp., Armonk, NY, USA).

The study was approved by the Institutional Ethics Committee, PGIMER, Chandigarh, India, with reference number NK/7986/Study/155.

Study setup

**Figure 1:**
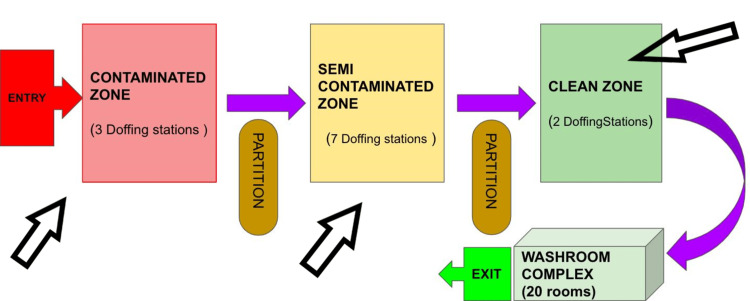
Sketch diagram of the segmented doffing corridor

**Figure 2:**
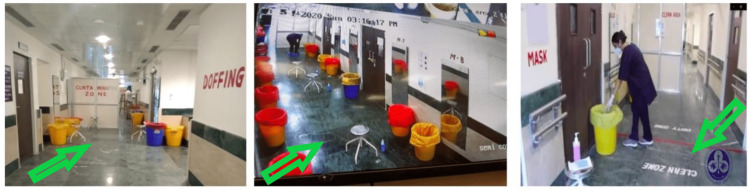
Real-time image of the doffing corridor in the COVID-19 hospital

The three-segmented zones of Doffing Corridor at the COVID-19 hospital facility of PGIMER, Chandigarh, India, were named (a) Contaminated zone (for removal of the outer set of gloves, gown, face shield, and leg cover); (b) Semi-contaminated zone (for removal of the jumpsuit and cap). Shoe cover being removed while moving from semi-contaminated to clean area (c) Clean zone (removal of N95 masks). Further, each doffing zone was expanded to include many doffing stations. The contaminated area had three doffing stations, the semi-contamination zone had seven doffing stations, and the clean area had two doffing stations. The doffing corridor was accessorized with multiple full-length mirrors, rotating stools, alcohol hand rubs fitted to dispensers, doffing steps chart fixed on each wall and biomedical waste bins. The doffing corridor was equipped with four real-time surveillance cameras (one in the contaminated zone, two in the semi-contaminated zone, and one in the clean zone) with an audio command system to guide doffing in each zone. A sketch diagram of the segmented doffing corridor is shown in Figure 1, and the real-time image of the doffing corridor is shown in Figure 2.

## Results

**Figure 3:**
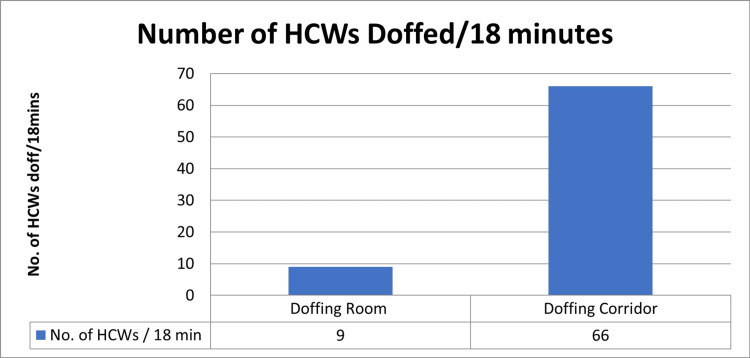
Number of doffing events by HCWs in the doffing corridor vs. the doffing room in the same period of time HCWs: healthcare workers

Based on the time taken for doffing, it was found that in 18 minutes, 66 doffing events were completed in the doffing corridor. However, in a doffing room a maximum of nine doffing events were conducted in the same time interval. The average time taken for doffing was around seven minutes in the doffing room. In contrast, the average time taken for the doffing in the doffing corridor was about 3.5 minutes. The most common errors made by the HCWs during the doffing steps were noted, and necessary corrections were made to reduce the mistakes [9]. In Figure 3, the histogram shows the number of doffing events done by the HCWs in the doffing corridor vs. doffing room in the same period of time. 

**Figure 4:**
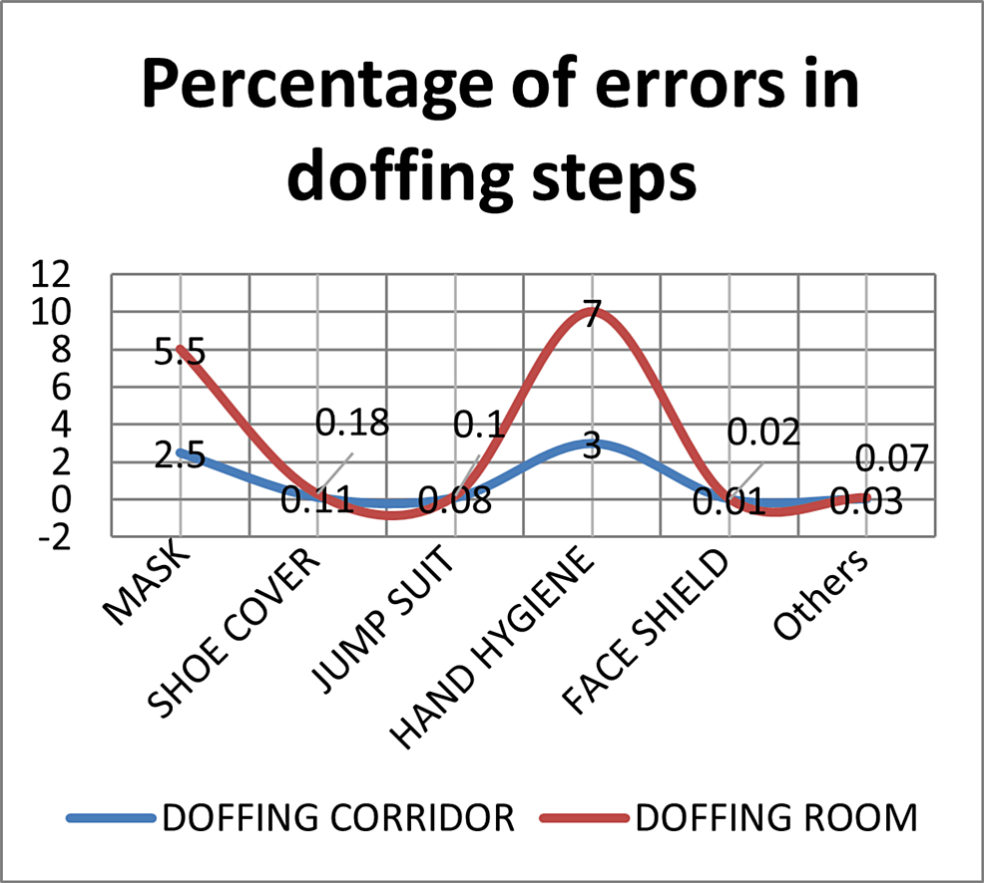
Comparison of percentage of errors during doffing steps in the doffing corridor vs. doffing room

The errors in the doffing steps were calculated in the doffing room and the doffing corridor, respectively. The errors in doffing steps were calculated as 5.5% and 2.5%, respectively, in the event of the removal of masks, 0.25% and 0.0125%, respectively, in the event of the removal of face shields, 0.18% and 0.11%, respectively, in the event of the removal of the shoe cover, 0.1% and 0.08%, respectively, in the event of the removal of the jumpsuit, and 7.0% and 3.0%, respectively, in the event of hand hygiene. Other errors included technical errors or lack of orientation to the doffing area (0.07% and 0.035%), respectively. The comparison of the errors in the doffing steps is shown in Figure 4. 

**Figure 5:**
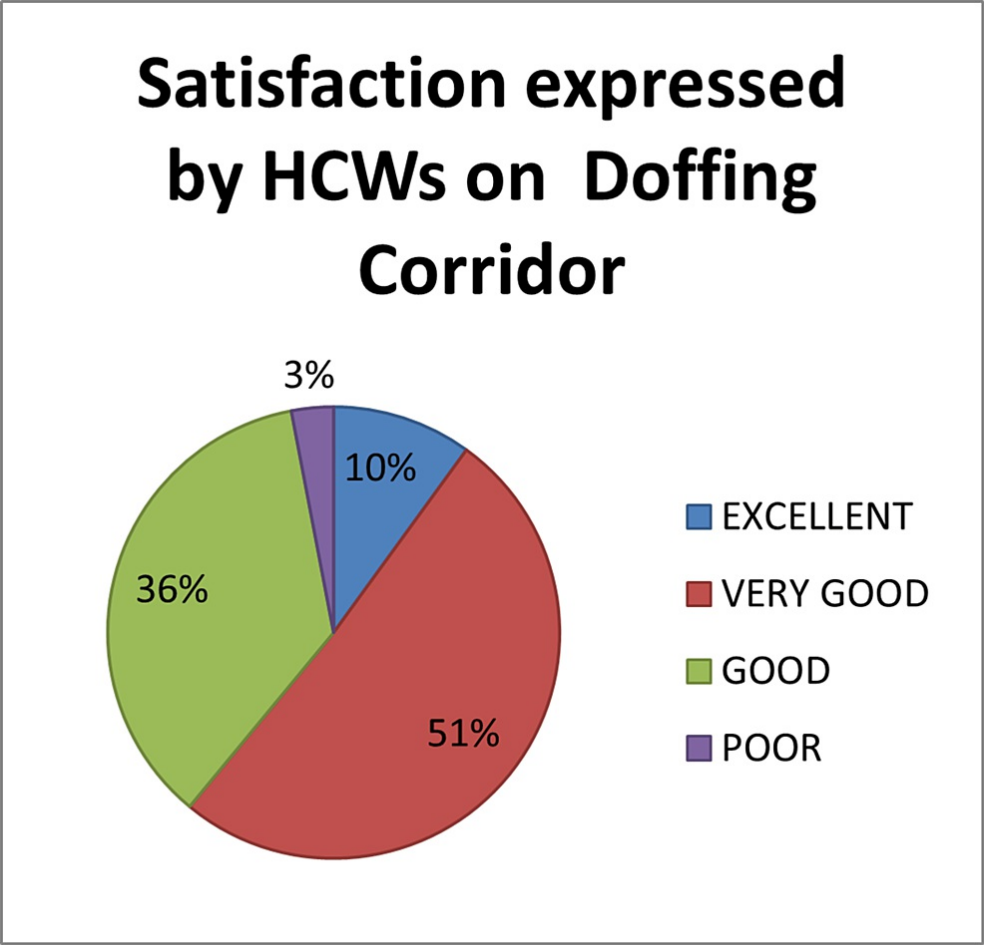
Pie diagram showing the percentage of satisfaction expressed by HCWs on doffing corridor Data collected from Epicollect 5 software HCWs: healthcare workers

Based on the four-grade rating system, 36% of HCWs rated the level of satisfaction as good, and 51% of the HCWs as very good for the doffing process in doffing corridors. A pie diagram (Figure 5) shows satisfaction expressed by HCWs on the doffing corridor.

In this study, 51 HCWs, out of 7885 HCWs who doffed in the doffing corridor, and 11 HCWs, out of 5142 HCWs who doffed in the doffing room, acquired COVID-19 infection. The odds ratio was calculated by taking the two groups of HCWs doffed in two different areas (doffing corridor and doffing room) by considering a confidence interval of 95% taking the steps of doffing as a risk factor. The result showed that the odds of HCWs who doffed in the doffing room were 3.03 times more likely to get COVID-19 infection than the odds of the HCWs who doffed in the doffing corridor [OR=3.03, CI (1.58-5.8), p<0.001], which was statistically significant as shown in Table 1.

**Table 1:** Shows the risk ratio (odds ratio) of the HCWs doffed in the doffing room and the doffing corridor HCWs: healthcare workers

	COVID +ve HCWs	COVID -ve HCWs	Odds Ratio (95% CI)	p-value
Doffing Room	11	5131	3.03(1.58-5.8)	<0.001
Doffing Corridor	51	7835	1	-

## Discussion

To the best of our knowledge, the current study on the innovative doffing corridor to curb COVID-19 spread is first of its kind.

The PDSA cycle, i.e., Plan-Do-Study-Act for the doffing corridor, was performed, and necessary modifications were done after careful observation of doffing errors to reduce the errors in doffing events to increase the performance of the doffing corridor [10].

A previous study on the doffing of the PPE kit showed modifications to reduce physical stress when removing shoe covers and other items [11]. A similar study showed the impact of the environmental design of the doffing area on the process of ease and error-free doffing [12]. Another study showed replacement of consumable items was managed carefully to prevent negligence in doffing to prevent self-infection among healthcare workers [13]. A study found a strong association between hand hygiene compliance and adherence to PPE kits with COVID-19 infection [14].

Another study described the HCWs were the person to provide care to COVID-19 patients, and after their duty, there was a chance that they might spread the disease [15]. A previous study showed that most of the problems associated with the PPE kit occurred during the doffing process; hence the doffing process is given the most attention in this study [16]. A survey conducted in PGIMER showed 34% errors in hand hygiene and 8% errors in N95 mask removal during the doffing process, where the facility of the doffing corridor was used [17]. One interesting finding in the doffing corridor was that HCWs simultaneously doffing at a time, helping each other follow the audio commands, and reduced the mask removal error to 2.5%, which had similarities to a previous study [6]. The most common errors made by the HCWs during the doffing steps were noted down, and necessary corrections were made to reduce the mistakes [9].

Strength 

Separate doffing zones decreased the chance of exposure to the infected material as most infected outer layer was doffed in the first step, and the least infected material is removed in the last step in the clean zone. Thirteen people could doff at a time, making the process faster and safer. Real-time surveillance cameras and two-way communication systems helped the HCWs to guide in each step of the doffing hence decreasing the chance of errors in the steps of doffing.

Limitations

As the study design is an observational and prospective study involving a retrospective WHO questionnaire, there could be chances of observation and recall bias among HCWs. Due to the stress of working in the COVID-19 pandemic zone, the information collected from the HCWs could be over- or under-reported. The heavy-duty load of HCWs could also be a reason for over-reporting infection prevention and control (IPC) practices. The probability of exposure from the community could not be excluded from the study.

Recommendations

Correct steps of hand hygiene are recommended and followed in the study to prevent the spread of the infection. Announcement via speakers in each doffing step was religiously followed to remind the HCWs of hand hygiene. Rigorous and frequent training was the utmost mode to build up strength and confidence in HCWs to deal with the COVID-19 pandemic and to protect themselves from the infection.

## Conclusions

The newly evolved COVID-19 pandemic has challenged healthcare organizations to fight and curb infection using innovative technologies. Considering the fact that the infection rate was very high among HCWs, it was essential to safeguard the COVID-19 warriors. The innovative doffing corridor concept was the first of its kind to expedite the doffing process with minimum exposure to the contaminated PPE items. The current study was planned and executed in a tertiary care COVID-19 hospital of PGIMER, Chandigarh. The innovative doffing corridor method was outstanding compared to the doffing room concept. The key findings of this study such as high satisfaction rate, less exposure to the contagion, and heavy doffing using the doffing-corridor, which can be scaled up or down to make any hospital pandemic-ready in case of future emergency. 
